# Delayed Gastric Emptying After Sleeve Gastrectomy Is Associated with Poor Weight Loss

**DOI:** 10.1007/s11695-022-06323-2

**Published:** 2022-10-27

**Authors:** Anagi Chethana Wickremasinghe, Yazmin Johari, Cheryl Laurie, Kalai Shaw, Julie Playfair, Paul Beech, Helen Yue, Louise Becroft, Geoffrey Hebbard, Kenneth S. Yap, Wendy Brown, Paul Burton

**Affiliations:** 1 Department of Surgery, Central Clinical School, Alfred Centre, Monash University, Level 6, 99 Commercial Rd, VIC 3004 Melbourne, Australia; 2 Oesophago-Gastric and Bariatric Unit, Department of General Surgery, The Alfred Hospital, Melbourne, VIC 3004 Australia; 3 Department of Nuclear Medicine and PET, The Alfred Hospital, Melbourne, VIC 3004 Australia; 4 Department of Gastroenterology, Royal Melbourne Hospital and University of Melbourne, Parkville, VIC 3050 Australia; 5 Department of Medicine, Monash University, Alfred Hospital Campus, Melbourne, VIC 3004 Australia

**Keywords:** Sleeve, Physiology, Weight regain, Bariatric outcome, Clinical trial, Complications, Reflux, Gastric emptying, Nuclear scintigraphy, Diagnostic test, Physiological failure, Bariatric surgery mechanism

## Abstract

**Background:**

Intermediate to long-term weight regain is a major challenge following sleeve gastrectomy (SG). Physiological changes that mediate the extent of weight loss remain unclear. We aimed to determine if there were specific esophago-gastric transit and emptying alterations associated with weight regain.

**Material and Methods:**

Participants greater than 12 months post-SG were categorized into optimal (*n* = 29) and poor weight loss (PWL) (*n* = 72). All patients underwent a liquid contrast barium swallow demonstrating normal post-surgical anatomy and a protocolized nuclear scintigraphy designed specifically to characterize gastric emptying following SG.

**Results:**

The %total weight loss in the optimal group was 26.2 ± 10.5 vs. 14.3 ± 8.8% in the PWL group (*p* = 0.001). Scintigraphy showed PWL had relatively increased gastric emptying half-time (GE ^1/2t^) 35 (IQR 23) min vs 19 (IQR 5.5) min (*p* = 0.001). The multivariate regressions delineated GE ^1/2t^ as the best diagnostic measure for PWL (OR 1.16; CI 1.04–1.29, *p*-value 0.021). The probability of PWL increased by 16% for every 1-min increase above 21 min of GE ^1/2t^. A threshold of 21 min was found to have 88% sensitivity and 69% specificity predicting poor weight loss.

**Conclusion:**

Gastric emptying half-times greater than 21 min appear to reliably correlate with poor weight loss following SG. Additionally, further elevations above 21 min in emptying half-time increase the risk of poor weight loss. We have shown nuclear scintigraphy represents a simple and accurate diagnostic tool in patients who experience poor weight loss after SG, provided substantially altered reporting references in interpreting nuclear scintigraphy are applied.

**Graphical abstract:**

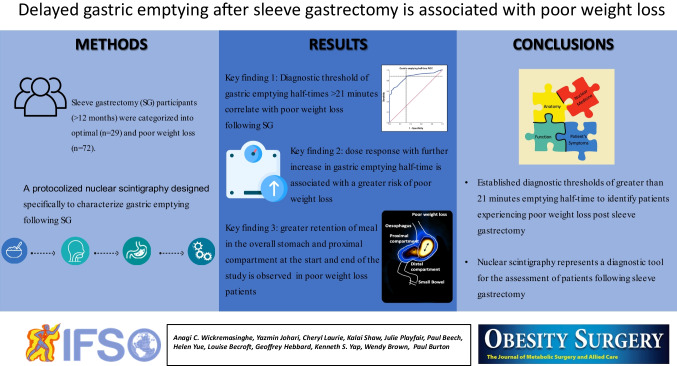

## Introduction

Intermediate to longer term weight regain has emerged as a principal challenge following sleeve gastrectomy (SG), with reports of this occurring in 6–76% of patients after 2 to 6 years [1–7]. Longer term studies have identified re-operation rates of 30% for weight regain [8]. This is of substantial significance as sleeve gastrectomy (SG) is the most common bariatric surgical procedure performed worldwide and achieves excellent initial weight loss outcomes [9, 10].

Presently, understanding of the key physiological changes mediating both initial weight loss and those associated with weight regain is limited. This has constrained the development of tailored diagnostic tests that can accurately assess whether a physiological problem with the sleeve itself has led to weight loss failure.

Proposed mechanisms of weight regain following SG primarily relate to anatomical change and luminal dilatation [11, 12]. Associations include technical factors relating to initial sleeve size bougie size, presence of a fundal remnant, and extent of antral resection. Ghrelin levels, follow-up support, and lifestyle and behavior change could also be relevant but are difficult to quantify.

It is likely that disruption of aspects of the physiology critical to weight loss is a key cause of weight regain or poor weight loss. Accelerated gastric emptying is common after SG [13–15]. A model of esophageal-mediated transit facilitates the rapid gastric emptying and involves esophageal-mediated isobaric pressurization in the vertical compartment along with repeat peristalsis. The non-compliant antrum then contracts in response, reflexively facilitating trans-pyloric flow [16]. However, translation into understanding of mechanisms specifically correlating with weight loss or useful diagnostic tests has not yet been achieved.

We hypothesized that variations in esophageal and gastric transit from the expected sleeve gastrectomy state would be associated with weight regain and could characterize physiological failure of the procedure. Our primary aim was to determine whether:

- Specific physiological alterations are associated with weight regain and can be identified using a standardized nuclear scintigraphy and interpretation protocol
- Identified variables could be translated into a simple, reliable diagnostic test predictive of physiological failure of the sleeve gastrectomy

## Methods

### Participants

Eligible participants were a minimum of 12 months post-operative with a diagnosis of poor weight loss. Poor weight loss was defined as those with < 15% total body weight loss (TBWL), < 40% excess weight loss (EWL), or < 20 kg weight loss who presented to our clinic for evaluation of weight regain and inadequate weight loss. The optimal group consisted of patients with good weight loss (> 30 kg or > 20% of TBWL or > 50% EWL). Participants were excluded from the study if they were pregnant or breastfeeding, had previous esophago-gastric surgery, or had substantial adverse symptoms requiring further investigations.

All patients had undergone liquid contrast swallow and gastroscopy as part of their post-operative assessment of weight loss and were not deemed to have demonstrated significant gastric dilatation or distortion on those investigations. Additionally, the surgeon undertook a detailed clinical assessment and structured interview to ensure that there was appropriate knowledge of the necessary behavioral change required and that eating patterns were reasonable. Ethics approval was obtained from the Alfred Human Research and Ethics Committee (HREC) no. 380/16 and 185/20. Informed written consent was obtained before the commencement of the study.

### Nuclear Medicine Scintigraphy Studies

A standardized esophageal transit and gastric emptying study was performed [17]. Participants fasted overnight (at least 6 h of fasting) prior to the procedure.

#### Meal

Participants consumed a radiolabeled semi-solid porridge meal. The meal consisted of 30 g instant porridge, 100 mL full cream milk, one teaspoon of sugar, and 40 MBq of Tc-99 m Calcium Phytate (Austin Health, Melbourne, Australia).

#### Scintigraphy Technique

Nuclear scintigraphy was performed using a Siemens Symbia™ Evo Excel Gamma Camera. All images were processed on a General Electric Xeleris ™ Functional Imaging Workstation version 4.

#### Esophageal Transit

The first part of the esophageal assessment included two semi-solid swallows taken at the commencement of the meal. Patients were asked to swallow a three-quarter tablespoon of semi-solid porridge in one attempt in the standing position. The second part of the esophageal assessment was two liquid swallows which were performed at the end of the gastric emptying. Patients swallowed 10 MBq of Tc-99 m Calcium Phytate in 10 mL of water administered orally by a syringe while in the supine position. Images were acquired at 1 s per frame for 60 s in the posterior projection for each swallow.

#### Gastric Emptying

Following the first part of the esophageal assessment, patients consumed the remaining meal within 5 min. Patients were then imaged supine in the left anterior oblique 30° projection, and images were taken 5 s per frame for 90 min.

#### Image Processing and Analysis

For gastric emptying and esophageal transit studies, regions of interest (ROIs) were drawn around the esophagus, neo-stomach (including proximal and distal stomach), and small bowel. The overall gastric emptying half-time was calculated as the time required by the stomach ROI to empty 50% of the ingested meal. Radioactive counts were represented as a function of time in a time-activity curve (TAC). The counts in the first and last frames of each anatomical region were expressed as a proportion of the counts in each ROI over total counts. Additionally, the sleeve shapes were classified into three different patterns of intragastric meal distribution: proximal (dilated portion of the proximal sleeve), distal (dilated portion of the antrum), and uniform (tubular-shaped sleeve).

### Statistical Analysis

Statistical analysis was performed using SPSS version 28 (SPSS Inc., Chicago, IL, USA) and GraphPad Prism version 9.1.2 (GraphPad Software, San Diego, CA, USA). Continuous parametric variables were presented as means and standard deviation, while non-parametric data were presented as median and interquartile range (IQR). The Mann–Whitney *U* test was used to compare non-parametric continuous variables, while categorical data were analyzed using Fisher’s exact test and presented as percentages. Univariate binary logistic regression was performed to identify the relationship between each variable and the outcome with multicollinearity being considered. Any confounding variables were adjusted for the multivariate binary logistic regression model with stepwise backward (Wald). Receiver-operating characteristic (ROC) curves were created to determine thresholds that discriminate poor weight loss. The area under the curve (AUC) was classified as follows: AUC > 9 = outstanding, 0.8–0.9 = excellent, 0.7–0.8 = acceptable, and < 0.7 = poor discrimination [18]. A two-sided *p*-value of 0.05 was considered statistically significant.

## Results

### Patient Demographics

Demographics and post-operative outcomes are summarized in Table 1. There were 29 patients in the optimal group and 72 patients in the poor weight loss group presenting for evaluation of weight regain following SG. As an additional control, pre-operative obese controls (*n* = 11) were compared. Pre-operative BMI was comparable between the optimal group (47.73 ± 10.89 kg/m^2^) and those with poor weight loss (46.44 ± 10.19 kg/m^2^), *p* = 0.572. The optimal group had achieved greater weight loss than the poor weight loss: %TWL 26.18 ± 10.45% vs. 14.25 ± 8.82% (*p* = 0.001). The nadir weights were comparable between the optimal and poor weight loss groups, 94.6 ± 18.5 kg vs 100.8 ± 21.5 kg (*p* = 0.190). On average, the poor weight loss had gained 12.3 ± 13.3 kg from nadir weight.

**Table 1:** Baseline demographics of patient groups

	Pre-operative obese controls (*n* = 11)	Optimal group (*n* = 29)	Poor weight loss (*n* = 72)	*p*-value
Age (years)	40.7 ± 13.0	47.73 ± 10.89	46.44 ± 10.19	0.572^a^
Female gender, *n* (%)	10 (90.9)	21 (73.33)	40 (59.71)	0.025^a^
Pre-operative weight (kg)	121.0 ± 16.3	129.37 ± 19.29	130.47 ± 24.43	0.828^a^
Pre-operative BMI (kg/m^2^)	45.8 ± 6.6	46.81 ± 6.85	47.97 ± 8.43	0.514^a^
Weight at follow-up (kg)	-	93.72 ± 18.79	111.57 ± 21.02	0.001^b^
BMI at follow-up (kg/m^2^)	-	33.95 ± 7.70	41.00 ± 7.40	0.001^b^
EWL (%)	-	64.71 ± 29.29	32.05 ± 26.38	0.001^b^
TWL (%)	-	26.18 ± 10.45	14.25 ± 8.82	0.001^b^

Values expressed as mean ± SD

^a^*p*-value calculated using one-way ANOVA for multiple comparisons

^b^*p*-value calculated using Student’s *t* test

### Esophageal Bolus Clearance

**Fig. 1:**
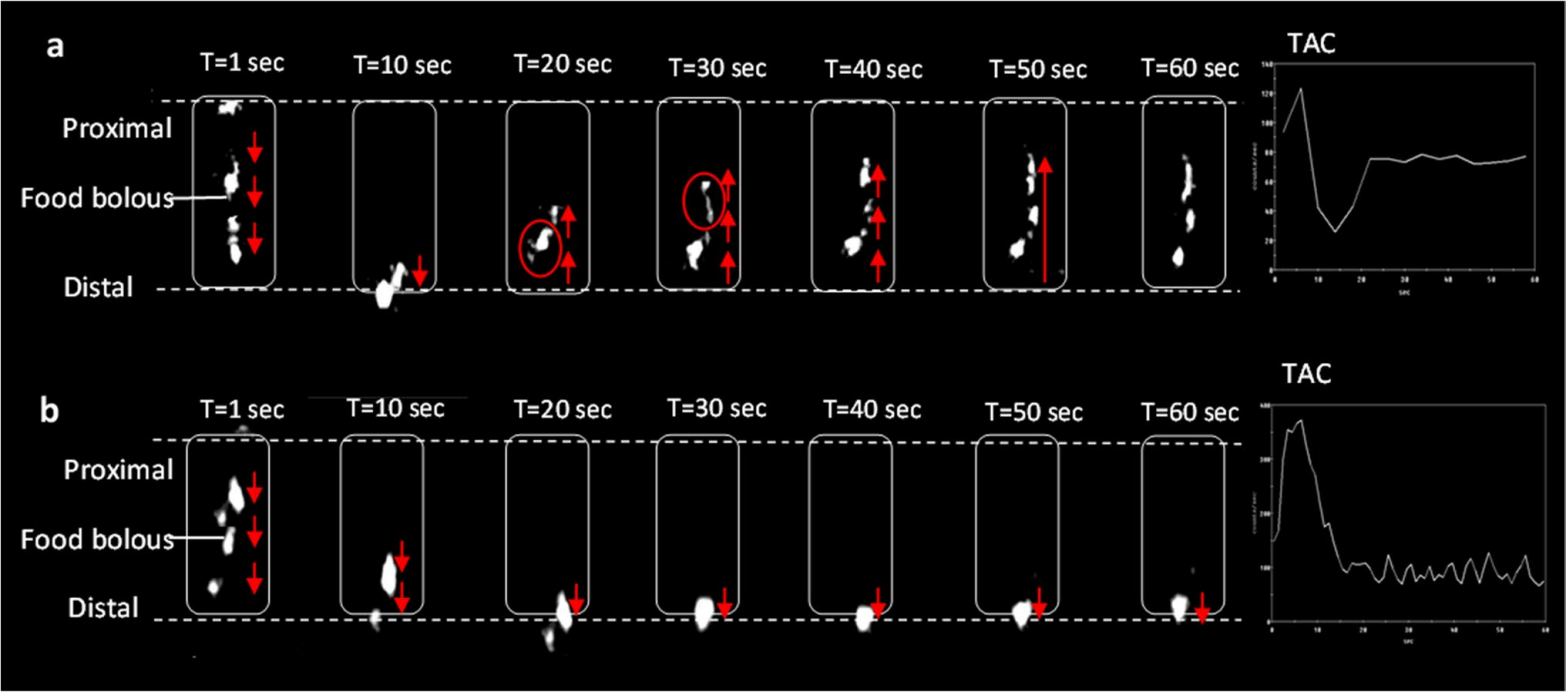
Bolus reflux on 60-s nuclear scintigraphy esophageal swallow study. Upper dotted line = manubrium, lower dotted line = xiphisternum. **a** Bolus-induced deglutitive reflux pattern. **b** No reflux

Both optimal and poor weight loss groups had similar esophageal transit results for liquid and semi-solid swallows. Two optimal and four poor weight loss patients experienced a delay during liquid swallows (*p* = 0.999). There was a reduction in the incidence of triggered deglutitive reflux in poor weight loss group compared to the optimal group (liquids: 31.3% vs 75.9%, *p* = 0.006; semi-solids: 22.4% vs 75.9%, *p* = 0.001) (Fig. 1a).

### Intragastric Meal Distribution

**Fig. 2:**
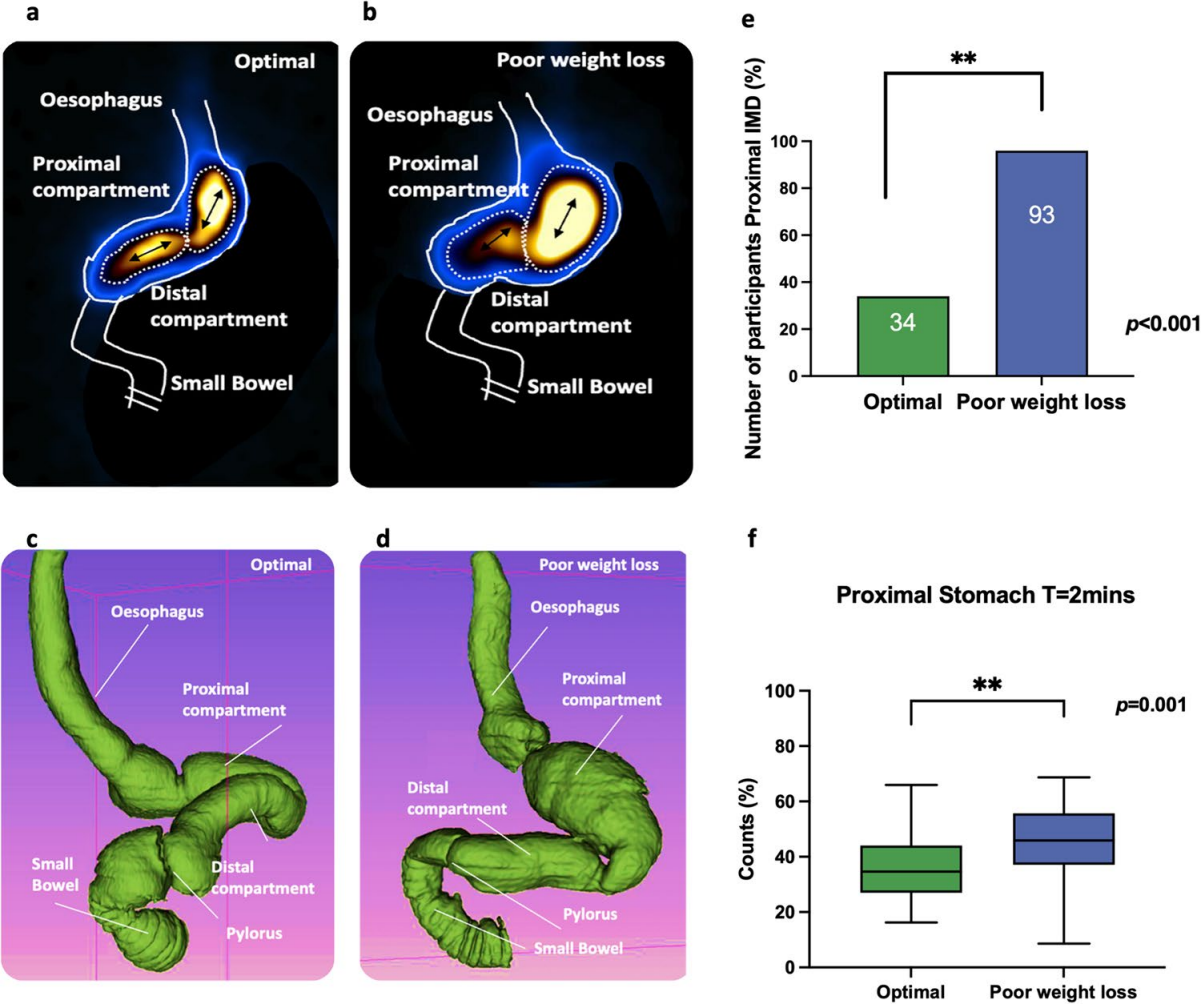
Intragastric meal distribution (IMD) illustration in optimal sleeves compared to poor weight loss patients Panels **a** and **b** are illustrative drawings demonstrating nuclear scintigraphy images with presumed anatomy and regions of interest (ROI) as drawn. **c** and **d** illustrate 3D reconstruction volumetric computed tomography of the gastric sleeve. **e** Number of participants with a proximal IMD. **f** Proportion of counts in the presumed proximal stomach

Upon ingesting the radiolabeled meal, three different patterns (proximal, distal, and uniform) of intragastric meal distribution were noted at the start of the scan (Fig. 2). The initial localization of the semi-solid meal was uniformly concentrated in the majority 65.5% (*n* = 19) of the optimal group in comparison to the 6.0% (*n* = 4) poor weight loss group (*p* < 0.0001). Semi-solid concentrate was primarily found in the proximal region of the stomach in the poor weight loss 86.11% (*n* = 62) compared to 34.5% (*n* = 10) of the optimal group (*p* < 0.0001). Quantitative measurement of intragastric meal distribution showed a significant increase in the proportion of counts in the proximal stomach of the poor weight loss compared with the optimal group, 46.38% (IQR 18.28%) vs. 34.66% (IQR 17.12%), *p* = 0.014.

### Gastric Clearance and Intestinal Delivery

The emptying half-time rates significantly differed among the two groups (Table 2), with emptying times delayed in the poor weight loss group. Emptying half-times as follows: gastric 35 (IQR 23) min vs.19 (IQR 5.50) min, *p* = 0.001, proximal 33 (IQR 22) min vs. 19 (IQR 5.75) min, *p* = 0.001, and distal 35 (IQR 28) min vs. 20 (IQR 6.75) min, *p* = 0.001.

**Table 2:** Nuclear scintigraphy

	Optimal group (*n* = 29)	Poor weight loss (*n* = 72)	*p*-value^a^
Esophageal transit study			
Delay in transit of liquid, *n* (%)	2 (6.9)	4 (6.0)	0.999
Delay in transit of semi-solids, *n* (%)	2 (6.9)	5 (7.5)	0.999
Deglutitive reflux of liquids, *n* (%)	22 (75.9)	21 (31.3)	0.006
Deglutitive reflux of semi-solids, *n* (%)	22 (75.9)	15 (22.4)	0.001
Gastric transit study			
Gastric emptying half-time, median (IQR) minutes	19 (5.50)	35 (23)	0.001
Proximal sleeve emptying half-time, median (IQR) minutes	19 (5.75)	33 (22)	0.001
Distal sleeve emptying half-time, median (IQR) minutes	20 (6.75)	35 (28)	0.001
*Proportion of counts at T* = *2 min, median (IQR)*			
Esophagus (%)	4.12 (4.7)	4.38 (2.89)	0.649
Overall stomach (%) Proximal (%) Distal (%)	53.18 (27.88) 34.66 (17.12) 19.72 (11.79)	69.84 (14.17) 46.38 (18.28) 20.57 (13.38)	0.003 0.014 0.252
Small Bowel (%)	40.66 (28.97)	24.64 (14.89)	0.002
*Proportion of counts at T* = *90 min, median (IQR)*			
Esophagus (%)	0.39 (0.24)	0.82 (0.63)	0.240
Overall stomach (%) Proximal (%) Distal (%)	3.79 (4.35) 1.93 (2.85) 1.53 (2.04)	10.21 (12.82) 6.72 (8.49) 3.70 (3.75)	0.001 0.001 0.462
Small Bowel (%)	95.84 (4.39)	89.22 (13.30)	0.001

^a^*p*-value calculated using Student’s *t* test

**Fig. 3:**
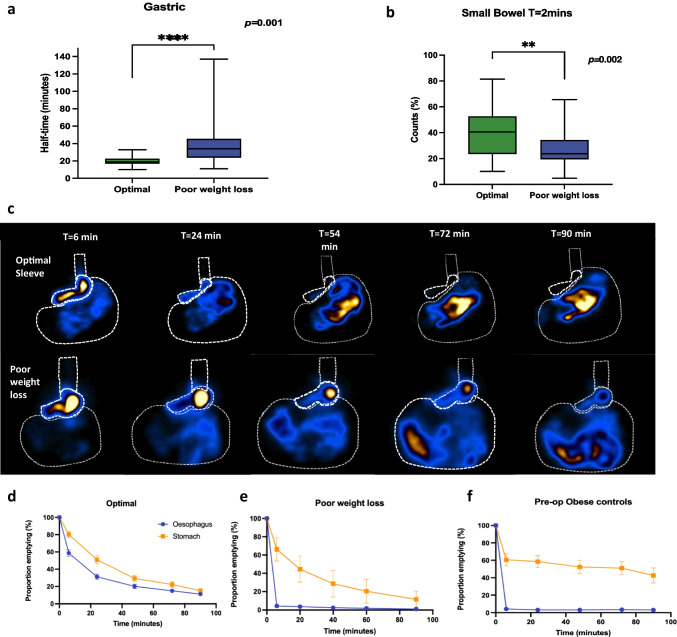
Nuclear scintigraphy: gastric clearance and intestinal delivery. **a** Gastric emptying half-time. **b** Proportion of counts in the small bowel within the first 2 min of image acquisition. **c** Schematic representation of proportional emptying in optimal sleeves and poor weight loss. **d**–**f** Emptying of the esophagus and stomach optimal sleeves, poor weight loss, and pre-operative obese controls over the 90-min study

On the initial acquisition frame (*T* = 2 min), a significant proportion of radioactive meal hyper-accelerated into the small bowel in the optimal group compared to the poor weight loss group 40.66 (IQR 28.97) % vs. 24.64 (IQR 14.89) %, *p* = 0.002 (Fig. 3b). At the end of the scan, the poor weight loss group had a significant amount of meal retained in the stomach 10.21 (IQR 12.82) % compared to the 3.79 (IQR 4.35) % of the optimal group, *p* = 0.001. The majority of the meal was located in the proximal part of the stomach 6.72 (IQR 8.49) % vs. 1.93 (IQR 2.85) %, *p* = 0.001.

### Co-dependent Clearance

In the optimal group, the esophagus and stomach emptied in a similar pattern, i.e., they demonstrated to emptying at an equivalent rate (Fig. 3d). However, the poor weight loss group did not appear to empty in this manner where the esophagus and stomach emptied as two distinct compartments, similarly to the way pre-operative participants with an intact stomach emptied.

### Risk Factors for Poor Weight Loss After Sleeve Gastrectomy

Table 3 shows the physiological variables evaluated for their contribution to poor weight loss after sleeve gastrectomy. The significant univariate analysis factors included gastric emptying factors; gastric emptying rate (OR 1.21; CI 1.10–1.34), proximal emptying rate (OR 1.15; CI 1.07–1.24), distal emptying rate (OR 1.16; CI 1.07–1.25), proportion of counts overall stomach at *T* = 2 min (OR 1.05; CI 1.02–1.08), proportion of counts overall stomach at *T* = 90 min (OR 1.23; CI 1.09–1.38), proportion of counts proximal stomach at *T* = 2 min (OR 1.05; CI 1.01–1.08), proportion of counts proximal stomach at *T* = 90 min (OR 1.20; CI 1.06–1.36), proportion of counts small bowel at *T* = 2 min (OR 0.95; CI 0.92–0.98), post-prandial reflux of liquid (OR 6.88; CI 2.55–18.62), and semi-solid swallows (OR 10.90; CI 3.90–30.41).

**Table 3:** Logistic regression of physiological variables associated with poor weight loss after sleeve gastrectomy

Variable	Univariate			Stepwise multivariate *		
	OR	95% CI	*P*-value	OR	95% CI	*P*-value
Gastric emptying rate	1.21	1.10–1.34	< 0.001	1.16	1.04–1.29	0.021
Proximal emptying rate	1.15	1.07–1.24	< 0.001			
Distal emptying rate	1.16	1.07–1.25	< 0.001	1.11	1.00–1.22	0.042
Proportion of counts overall stomach (*T* = 2 min)	1.05	1.02–1.08	0.003			
Proportion of counts overall stomach (*T* = 90 min)	1.23	1.09–1.38	0.001			
Proportion of counts proximal stomach (*T* = 2 min)	1.05	1.01–1.08	0.009			
Proportion of counts proximal stomach (*T* = 90 min)	1.20	1.06–1.36	0.005			
Proportion of counts distal stomach (*T* = 2 min)	1.03	0.98–1.07	0.281			
Proportion of counts distal stomach (*T* = 90 min)	1.04	0.93–1.17	0.468			
Proportion of counts small bowel (*T* = 2 min)	0.95	0.92–0.98	0.001			
Liquid transit (delay)	1.17	0.20–6.76	0.863			
Liquid transit (reflux)	6.88	2.55–18.62	< 0.001			
Semi-solid (delay)	0.92	0.17–5.03	0.922			
Semi-solid (reflux)	10.90	3.90–30.41	< 0.001			

^*^Stepwise backward (Wald) multiple regression was performed adjusting for proportion of counts in the overall stomach, proximal and distal stomach at *T* = 90 min

Emptying rates (total gastric and distal) were significant independent predictors of poor weight loss in the multivariate analysis. This model was found to be statistically significant (chi-square 40.991, *p* < 0.001), whereby the model explained 50.5% (Nagelkerke R-square) of the variance in poor weight loss outcomes following sleeve gastrectomy.

The probability of poor weight loss increased by 16% for every 1-min increase above 21 min of gastric emptying rate (OR 1.16; CI 1.04–1.29) and an 11% increase for every 1 min in distal emptying rate (OR 1.11; CI 1.00–1.22) after adjusting for proportion of counts in the overall stomach, proximal and distal stomach at *T* = 90 min. Therefore, there was a dose–response relationship with a higher probability of poor weight loss, the higher the gastric emptying half-time. For example, a patient with an emptying half-time of 40 min had a greater chance of poor weight loss (30.4%) compared to a patient with 30 min (12.8%) and a patient with 25 min emptying half-time (5%).

### Diagnostic Nuclear Scintigraphy Thresholds for the Assessment of Sleeve Gastrectomy Patients

**Fig. 4:**
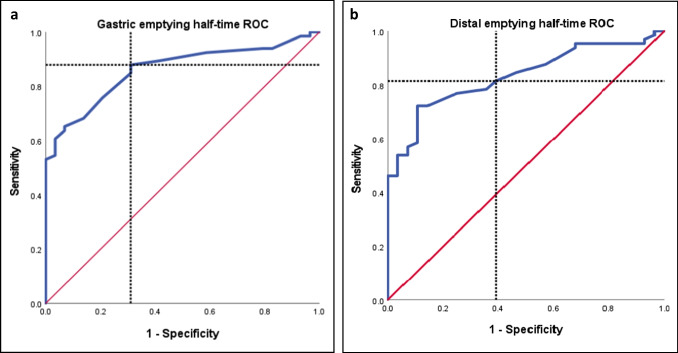
Diagnostic performance measures of poor weight loss using receiver-operating curves (ROC). Sensitivity and specificity illustrated by dotted line intersection. **a** ROC curve for gastric emptying half-time. AUC = 0.860 (95% confidence interval 0.787–0.933), *p* < 0.0001. Threshold set at 20.50 min, sensitivity = 87.9% and specificity = 69%. **b** ROC curve for proximal emptying half-time. AUC = 0.796 (95% confidence interval 0.706–0.886), *p* < 0.0001. Threshold set at 20.50 min, sensitivity = 80% and specificity = 60.7

The most highly discriminatory measures were overall gastric and distal emptying half-times. For gastric emptying half-time, a threshold of 20.50 min distinguished optimal weight loss patients from poor weight loss patients with 87.9% sensitivity and 69% specificity. The distal emptying rate was set at a threshold of 21.50 min with a sensitivity of 81.5% and specificity of 60.7%. The discriminant ability of the ROC curves was considered excellent for these measures (AUC = 0.860 and 0.083, *p* < 0.0001) (Fig. 4).

## Discussion

Our data demonstrate that specific physiological changes characterize weight loss failure following SG, strongly implicating these as fundamental to the mediation of weight loss. Three key findings of the study were as follows: firstly, in patients with optimal weight loss following SG, gastric emptying half-time was very consistently recorded as 19 min. In comparison for patients in the poor weight loss group, gastric emptying half-time was prolonged (35 (IQR23) min). Secondly, a highly accurate value for the simple measure of gastric emptying half-time (21 min) was identified as a diagnostic threshold for physiological failure, with further increases in emptying half-time representing a greater risk of weight loss failure. Thirdly, other markers of delayed gastric transit and retention were observed, along with a significant reduction in the incidence of triggered deglutitive and post-prandial reflux events.

Twenty-one minutes as a gastric emptying half-time was found to have 88% sensitivity and 69% specificity as a diagnostic threshold for poor weight loss following SG. This reliably and objectively delineates the physiologically normally functioning gastric sleeve. From a practical perspective, emptying half-times greater than 30 min appear substantially abnormal, despite an emptying time of 40–70 min being the expected reference range for an anatomically normal stomach. Substantially, altered reporting references in interpreting nuclear scintigraphy following SG are required. That, however, should be achievable in any department equipped with standard nuclear medicine scanning, attesting to the practicality of the test.

A pattern of proximal meal retention characterized poor weight loss, with over 80% of poor weight loss patients demonstrating this appearance. This likely represents a situation where increased fundal compliance facilitates retention in the absence of marked anatomic distortion. This also highlights the capacity of nuclear scintigraphy as a highly sensitive investigation as even subtle increases in emptying half-time were found to be predictive of significant risk of weight loss failure.

Bolus-induced deglutitive reflux events with reflex peristaltic contractions occurring during liquid and semi-solid swallows are expected following SG. This is due to esophageal peristalsis inducing isobaric pressurization of the proximal compartment and is the subsequent mechanism means via which rapid gastric and early intestinal delivery is achieved. However, this was seen markedly less by those with poor weight loss and is not surprisingly associated with a reduction in the rate of gastric emptying. This could possibly indicate another marker of increased fundic compliance as it does not pressurize therefore not causing reflux. This is consistent with the disruption of physiological processes, which appear essential to the functioning of SG.

A strength of our study was the design, in which we recruited specific cohorts of patients and utilized a further control group of patients with obesity that had anatomically normal stomachs in order to examine physiological differences and establish a diagnostic test. This work has built on a series of prior dedicated studies which had interrogated the mechanisms associated with SG and described many of the relevant physiological associations [10, 16, 19]. Importantly a dose–response relationship was observed with further increases in gastric emptying above 21 min, demonstrating a significant incremental risk of weight loss failure. This further attests to the direct association and potential causative relationship between increases in GE half-time and physiological failure of the SG.

We were able to establish that a series of different measures all consistently pointed to hyper-accelerated transit, with rapid intestinal delivery being essential to mediating weight loss. Nuclear scintigraphy was found to be an easily applicable, practical investigation possessing far higher fidelity than either liquid contrast swallow or gastroscopy. Furthermore, it provides an objective, numerical quantification of the functioning of the gastric sleeve.

A key outcome of our study has been the provision of direct evidence regarding the mediation of weight loss by specific surgically induced alterations. This has not previously been demonstrated. These findings significantly implicate specific physiological processes in weight loss and provided direct evidence of their importance. Complex mechanisms such as alterations in entero-hepatic bile circulation, changes in the microbiome, or alterations in GI hormones are suggested as the direct mechanism of weight loss following SG [20, 21]. The link between surgically induced anatomical alteration and how that triggers direct mechanisms of weight loss remain unclear. It would now seem likely that the rapid intestinal delivery of content and removal of the storage function of the stomach are closely associated with activating those direct signaling pathways.

**Fig. 5:**
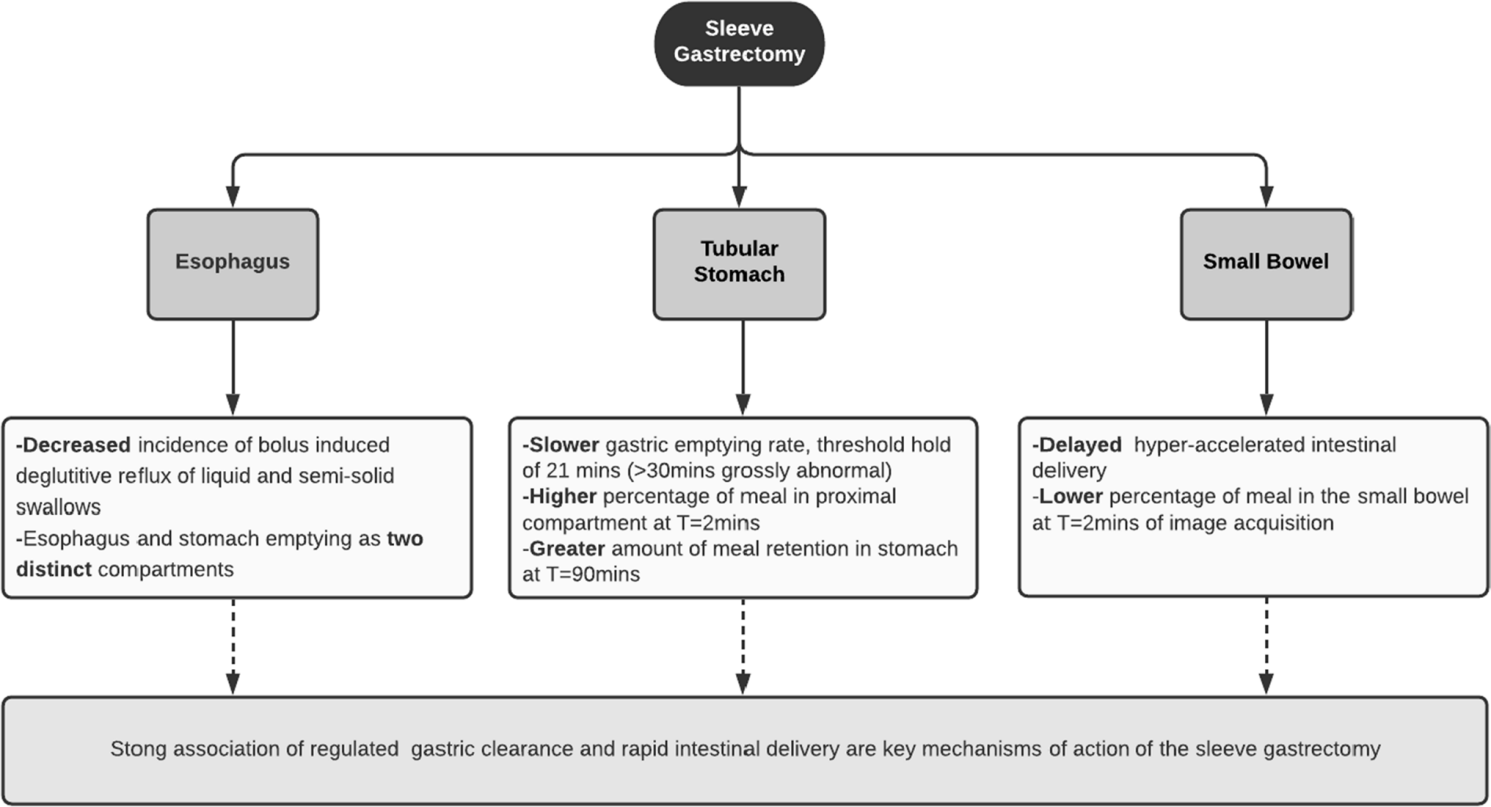
Summary of key pathophysiology of poor weight loss post-sleeve gastrectomy using nuclear scintigraphy

Future endeavors should be both mechanistic and clinical. Mechanistically, the correlation of these findings with other signaling perturbations that are proposed as the actual modulators of weight loss is important to understanding fundamental mechanisms of weight loss. Clinically, we would aim to evaluate the response of patients with elevated gastric emptying half-time to revisional surgery and hypothesize that these patients are more likely to respond (in our unit, a single anastomosis bypass is the preferred option). Additionally, the relevance of gastric emptying pre-operatively is worthy of consideration as this may play a role in the pathogenesis of obesity and represent a propensity to developing delayed gastric emptying and weight regain post-operatively. This study, would, however, require a large number of subjects tracked over a significant period of time, to be adequately powered (Fig. 5).

## Conclusion

The mechanisms of weight loss associated with SG appear significantly associated with rapid gastric transit and accelerated intestinal delivery. Variations from this physiology are consistent markers of weight regain and represent physiological failure of the procedure. These data have been translated to a simple diagnostic test to assess for physiological failure using nuclear scintigraphy, which requires only modifications to the interpretations and reporting reference ranges. This study has provided insights into the mechanism of weight loss following SG and identified a highly promising, objective, diagnostic test for patients presenting with weight regain, one of the key dilemmas following SG.
